# Clinical and demographic characteristics associated with the receipt of chemotherapy treatment among 7951 elderly metastatic colon cancer patients

**DOI:** 10.1002/cam4.143

**Published:** 2013-10-10

**Authors:** Emily S Reese, Eberechukwu Onukwugha, Nader Hanna, Brian S Seal, C Daniel Mullins

**Affiliations:** 1 Pharmaceutical Health Services Research Department, School of Pharmacy, University of MarylandBaltimore, Maryland; 2 Division of General and Oncologic Surgery, Department of Surgery, School of Medicine, University of MarylandBaltimore, Maryland; 3 Bayer Healthcare Pharmaceuticals, Inc.Whitehouse Station, New Jersey

**Keywords:** Chemotherapy, medicare, metastatic colon cancer, treatment

## Abstract

Among older individuals diagnosed with metastatic colon cancer (mCC) there is limited evidence available that describes the characteristics associated with advancing to second- and subsequent lines of treatment with chemotherapy/biologics. Our objective was to describe the trends and lines of treatment received among elderly mCC patients. Elderly beneficiaries diagnosed with mCC from 2003 to 2007 were identified in the Surveillance, Epidemiology and End Results (SEER)-Medicare dataset. Beneficiaries were followed up until death or censoring. Treatment lines were classified in combinations of chemotherapies and biologics. Modified Poisson regression was used to predict receipt of lines of treatment. Analyses controlled for age, race/ethnicity, gender, marital status, state buy-in during diagnosis year, SEER-registry site, Charlson comorbidity index (CCI), poor performance indicators, surgery of primary site, and surgery of regional/distal sites. Among 7951 Medicare beneficiaries identified with mCC, 3266 initiated therapy. Of these, 1440 advanced to second-line treatment. Of these, 274 advanced to a subsequent-line treatment. Surgeries of the primary tumor site and of the regional/distal sites and marital status were the most significant variables associated with advancing through second- and subsequent-line treatments. Greater than 80 years of age, African American race, SEER-registry area, less than 6 months state buy-in assistance in mCC diagnosis year, and having poor performance indicators were inversely associated with receipt of second- or subsequent-line treatments. Among elderly individuals diagnosed with mCC, we identified demographic, clinical, and regional factors associated with receipt of second- and subsequent-line chemotherapy/biologics. Additional research is warranted to understand the role of physician versus patient preferences as well as geographic differences explaining why patients advance through lines of chemotherapy.

## Introduction

Limited evidence exists that describes the factors associated with advancing of chemotherapy treatment lines in elderly metastatic colon cancer (mCC) patients. An even smaller amount of evidence exists that describes the correlates of initiation and additional lines of chemotherapy treatment in elderly mCC patients. Randomized controlled trials (RCTs) provide evidence that chemotherapy for advanced colon cancer is associated with improved outcomes and similar toxicity profiles across all age groups [1–10], despite increased costs [11]. The National Comprehensive Cancer (NCCN) guidelines recommend that patients with stage IV colon cancer be treated with some combination of chemotherapy with or without surgical resection [12]. Adherence to treatment guidelines is presumed to increase survival [13]. Using the National Cancer Database, researchers found NCCN guideline adherent treatment of stage IV colon cancer management adherence was 73%. NCCN guideline adherence was associated with patient age, comorbidity status, later year of diagnosis, and insurance status [14].

Evidence suggests that chemotherapy is effective in the elderly population (i.e., greater than 65 years of age) [15, 16], yet these patients are less likely to receive recommended therapy. Older patients are less likely to receive potentially curative treatments than younger patients. Chemotherapy is used less in patients older than 65 years of age, especially in nonwhite and in patients older than 75 years of age [17]. Community-dwelling patients greater than 75 years of age with surgical resection received less-toxic and shorter chemotherapy regimens than their younger counterparts and had fewer adverse events [18]. Elderly mCC patients exhibit age-related organ decline and multiple comorbidities, both of which increase concerns over the safety and the effectiveness of medical treatment; this perhaps causes physicians to be more conservative in the treatment of elderly patients. Prior studies, some of which used the Surveillance, Epidemiology and End Results (SEER)-Medicare dataset, document that at least one-half of elderly mCC patients do not receive any chemotherapy [19, 20], yet there is little evidence that describes treatment in this population. Our objective is to fill this void by identifying clinical and demographic factors associated with the initiation of chemotherapy treatment received among elderly mCC patients in hopes of drawing attention to the types of patients who are potentially undertreated. The evidence we generate can be used by clinicians and those who develop clinical guidelines to promote more standardized and evidence-based treatment recommendations for elderly mCC population in order to ensure appropriate, effective treatment, regardless of age.

## Methods

### Data source

This study uses the SEER dataset linked with Medicare (SEER-Medicare) claims to examine factors associated with receipt of subsequent lines of chemotherapy or biologics. Medicare administrative claims reflect fee-for-service reimbursement of medical treatment. The Medicare population includes individuals who are 65 years and older, in addition to those with chronic end-stage renal disease and individuals classified as disabled by the U.S. government. The SEER-Medicare dataset links Medicare claims data for patients to clinical measures upon a patient's entry into the SEER registry. Information available from the SEER-Medicare dataset not only includes information on clinical and demographic patient characteristics but also information about inpatient and outpatient stays, procedural and diagnoses codes and dates, chemotherapy administration, reimbursement amounts, home health care claims, hospice provider claims, and drug utilization [21].

### Study design and population

Elderly Medicare patients diagnosed with mCC between 2003 and 2007 were identified in the SEER-Medicare dataset. Associated Medicare claims were available from 2002 to 2009. Patients diagnosed with mCC were included in this study if they were eligible for the Medicare entitlement due to age (i.e., greater than 65 years of age) and had Medicare Parts A and B plans. Medicare Part A covers hospital and specialized care. Medicare Part B covers medically necessary services and preventive services. Individuals were excluded if enrollment into a managed care plan (Medicare Part C) took place in the year prior to their mCC diagnosis. These individuals were excluded because all claims for their care are not available from Medicare because a third party, managed care plan covers part of their health care expenses. Censoring occurred when the individual lost Parts A or B Medicare coverage or if the individual enrolled in a Medicare managed care plan.

Treatment lines were classified in combinations of chemotherapy and biologics. Investigators developed a series of rules in order to determine the sequence of treatment lines among mCC. This treatment line identification algorithm has been published elsewhere [22].

### Statistical analysis

Demographic variables including 5-year age group, race/ethnicity, gender, marital status, and months of state buy-in status were included in the model because of each variable's association with receiving chemotherapy. “Married” was defined as being married at time of mCC diagnosis as compared to individuals who are widowed, divorced, or never married were included in the “not married” group. The state buy-in co-insurance variable is a proxy for socioeconomic status. Clinical characteristics included in these analyses include Charlson comorbidity index (CCI), poor performance indicators (a composite of at least one of the following: use of walking aid, oxygen use, or wheelchair use), surgery of the primary site, and surgery of regional or distal sites. These are variables health care providers would take into consideration when deciding patient treatment. Each variable assessed was captured in the year prior to diagnosis. Descriptive statistics on demographic and clinical variables were generated for each treatment line identified. Chi-square tests compared each set of categorical variables. Modified Poisson regression was used to estimate risk ratio (RR) for treatment receipt associated with each covariate included in the regression models. This method was used because it estimates RRs and does not have convergence problems, as seen in the use of binomial regression [23]. Three separate models estimated the probability of entering into first-line, second-line, and subsequent-line treatment. Only individuals who were eligible for the outcome of interest (i.e., receipt of the various lines of treatment) were included in each model. Due to low sample size in some registry areas (San Francisco, Hawaii, New Mexico, Utah, Atlanta, San Jose, and rural Georgia), these sites were combined into “Other Registry Sites.” These analyses combined several proxies for poor performance status (use of walking aid, oxygen use, and wheelchair use) as a variable in the final models. All statistical analyses were conducted using SAS 9.2 software (Cary, NC).

## Results

**Figure 1:**
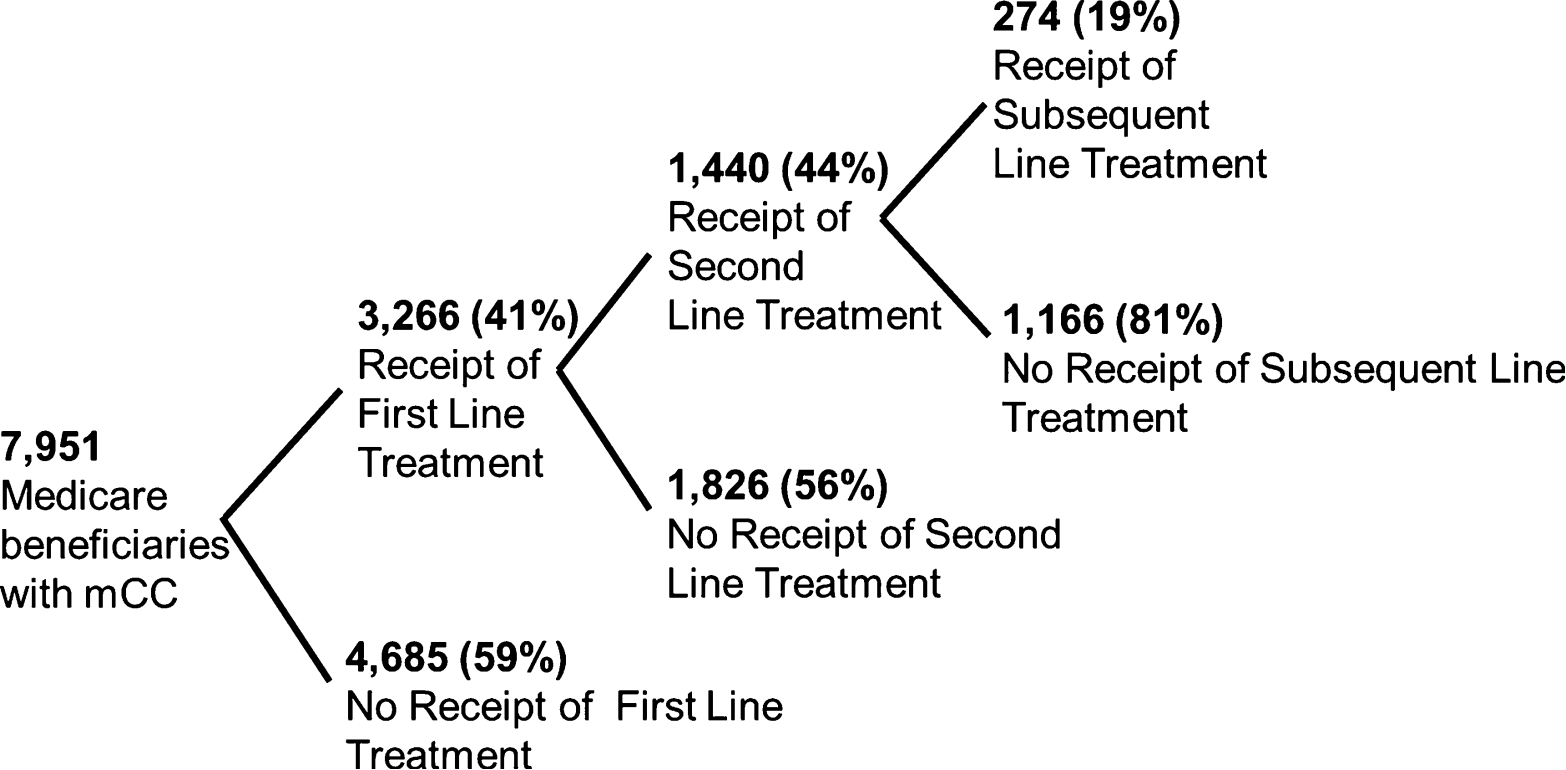
Proportion of Medicare beneficiaries diagnosed with metastatic colon cancer by lines of treatment.

As shown in Figure 1, among 7951 Medicare patients who were diagnosed with mCC, 3266 (41%) initiated therapy, while 59% did not receive any treatment with chemotherapy or biologics. Among those who initiated treatment, 1440 (44%) received a second-line treatment while only 274 (19%) of treated patients progressed to subsequent treatment beyond second-line treatment. The cohort was evenly distributed among age and gender groups. The population was predominantly white, non-Hispanic individuals and the majority did not have dual Medicare and Medicaid eligibility during the 12 months prior to mCC diagnosis. Clinically, the cohort had a low CCI and did not report a high prevalence of poor performance status indicators. More patients had surgery of the primary site and did not have surgery of regional or distal sites. Additional demographic and clinical characteristics of mCC Medicare beneficiaries by receipt of treatment lines are described in Table 1.

**Table 1:** Clinical and demographic characteristics of 7951 elderly mCC patients in the SEER-Medicare dataset

	First-line treatment (*n*=3266)			Second-line treatment (*n*=1440)			Subsequent-line treatment (*n*=274)		
			
	*N*	%	*P*-value	*N*	%	*P*-value	*N*	%	*P*-value
*Demographic characteristics*									
Age group									
65–69	748	22.90	<0.01	379	26.32	<0.01	73	26.64	<0.01
70–74	858	26.27		418	29.03		76	27.74	
75–79	869	26.61		383	26.60		72	26.28	
80+	791	24.22		260	18.06		53	19.34	
Race/ethnicity group									
African American	306	9.37	<0.01	122	8.47	<0.01	18	6.57	0.09
Hispanic	156	4.78		62	4.31		13	4.74	
White, non-Hispanic	2661	81.48		1191	82.71		230	83.94	
Another minority	143	4.38		65	4.51		13	4.74	
Sex									
Female	1660	50.83	<0.01	708	49.17	<0.01	140	51.09	0.25
Male	1606	49.17		732	50.83		134	48.91	
Marital status									
Married	1897	58.08	<0.01	905	62.85	<0.01	160	58.39	<0.01
Not married	1369	41.92		535	37.15		114	41.61	
Months of state buy-in during diagnosis year									
None	2830	86.65	<0.01	1266	87.92	<0.01	242	88.32	<0.01
1–6months	66	2.02		22	1.53		NR	NR	
7–12months	370	11.33		152	10.56		NR	NR	
*Clinical characteristics*									
Charlson comorbidity index									
0.00	2135	65.37	<0.01	975	67.71	<0.01	185	67.52	<0.01
1.00	736	22.54		312	21.67		66	24.09	
2+	395	12.09		153	10.63		23	8.39	
Poor performance indicators									
No	2968	90.88	<0.01	1340	93.06	<0.01	259	94.53	<0.01
Yes	298	9.12		100	6.94		15	5.47	
Surgery of primary site									
No	832	25.47	<0.01	301	20.90	<0.01	56	20.44	<0.01
Yes	2434	74.53		1139	79.10		218	79.56	
Surgery of regional or distal sites									
No	2636	80.71	<0.01	1165	80.90	<0.01	222	81.02	0.05
Liver metastatis surgery	468	14.33		211	14.65		35	12.77	
Other surgery	162	4.96		64	4.44		17	6.20	

mCC, metastatic colon cancer; SEER, Surveillance, Epidemiology and End Results; NR, cell values have been censored per SEER-Medicare Data Use Agreement to protect the privacy of human subjects.

### Factors associated with chemotherapy or biologic treatment

**Figure 2:**
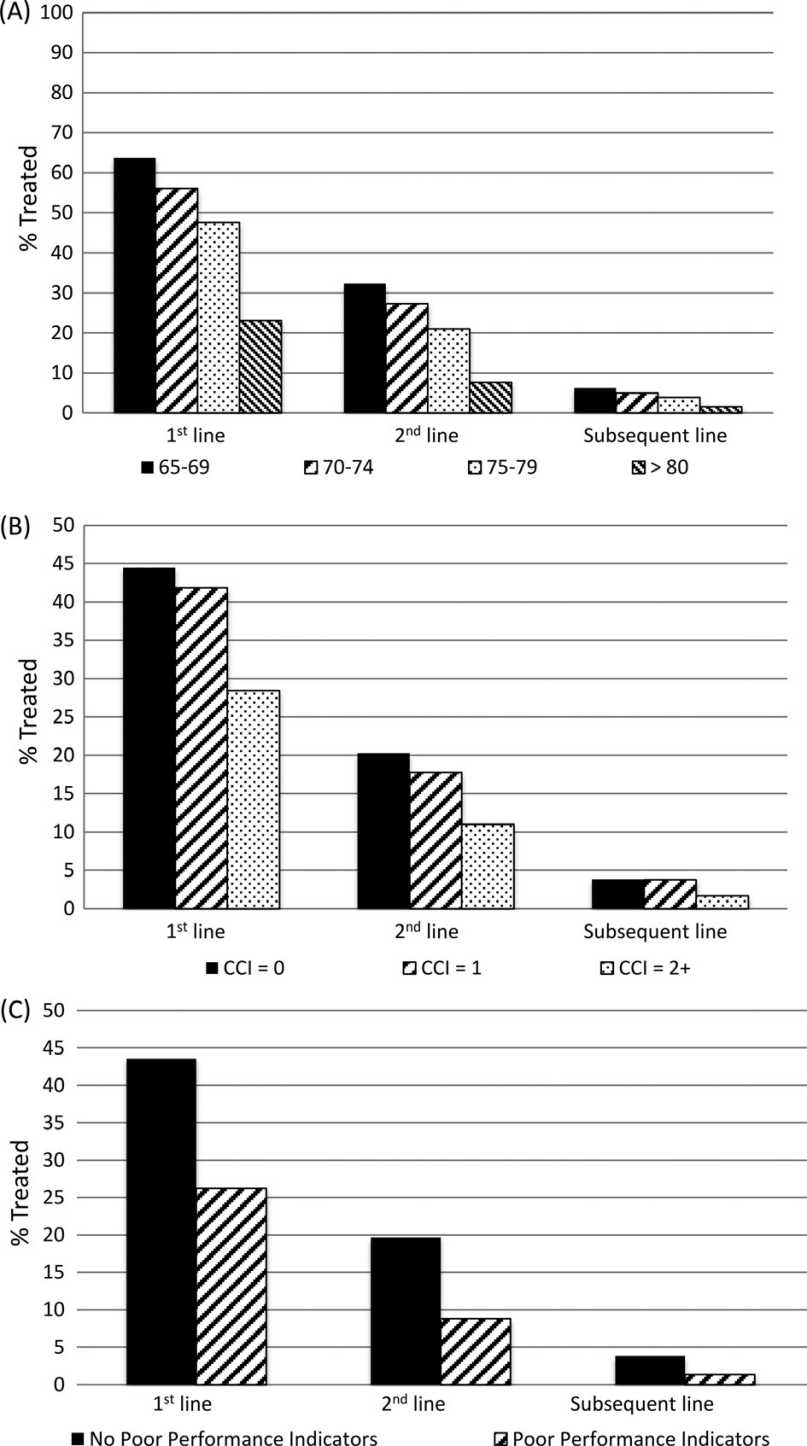
Percent of age group (panel A), Charlson comorbidity index (CCI) (panel B), and poor performance status indicators (panel C) by lines of treatment out of the entire cohort.

Receipt of surgery of the primary site and marital status were associated with receiving first-line chemotherapy (RR: 1.65, *P* < 0.0001 and RR: 1.29, *P* < 0.0001, respectively). Having liver metastasis surgery was also a statistically significant factor associated with initial chemotherapy treatment (RR: 1.17, *P* < 0.001) (Table 2). Results indicated differences between age groups, CCI, and poor performance status indicators among lines of treatment. Individuals in the oldest age group (>80 years), those with CCI of 2+, and those with poor performance status indicators (i.e., use of oxygen, a walking aid, or wheelchair) were less likely to be treated compared to their grouped counterparts. These discrepancies are shown in Figure 2.

**Table 2:** Regression results showing predictors of second- and subsequent-line treatments in patients with metastatic colon cancer in the SEER-Medicare dataset

	First-line treatment		Second-line treatment		Subsequent-line treatment	
			
	Risk ratio	95% confidence interval	Risk ratio	95% confidence interval	Risk ratio	95% confidence interval
Age group						
65–69	Reference		Reference		Reference	
70–74	0.90***	(0.85, 0.95)	0.87**	(0.77, 0.969)	0.790	(0.58, 1.08)
75–79	0.79***	(0.74, 0.84)	0.70***	(0.62, 0.783)	0.643***	(0.47, 0.89)
>80	0.43***	(0.40, 0.46)	0.29***	(0.25, 0.335)	0.288***	(0.20, 0.42)
Race/ethnicity						
White, non-Hispanic	Reference		Reference		Reference	
Another minority group	0.91	(0.80, 1.03)	0.87	(0.69, 1.09)	1.05	(0.58, 1.91)
African American	0.89**	(0.82, 0.98)	0.79***	(0.66, 0.93)	0.57**	(0.35, 0.92)
Hispanic	0.99	(0.88, 1.11)	0.84	(0.67, 1.06)	1.00	(0.57, 1.75)
Gender						
Male	Reference		Reference		Reference	
Female	1.03	(0.98, 1.08)	1.02	(0.93, 1.11)	1.05	(0.83, 1.34)
Marital status						
Not married	Reference		Reference		Reference	
Married	1.29***	(1.22, 1.36)	1.46***	(1.32, 1.61)	1.20	(0.93, 1.55)
SEER-registry area						
Connecticut	Reference		Reference		Reference	
Detroit	0.81***	(0.72, 0.91)	0.75***	(0.61, 0.92)	1.04	(0.62, 1.77)
Iowa	0.84***	(0.74, 0.94)	0.66***	(0.54, 0.82)	0.58*	(0.31, 1.08)
Seattle	0.85**	(0.75, 0.97)	0.71**	(0.57, 0.90)	0.88	(0.48, 1.60)
Los Angeles	0.99	(0.88, 1.11)	0.95	(0.77, 1.17)	0.98	(0.54, 1.75)
Greater California	0.83***	(0.75, 0.92)	0.67***	(0.56, 0.80)	0.76	(0.47, 1.23)
Kentucky	0.81***	(0.72, 0.90)	0.54***	(0.43, 0.67)	0.44**	(0.24, 0.84)
Louisiana	0.76***	(0.67, 0.87)	0.64***	(0.51, 0.80)	0.67	(0.36, 1.25)
New Jersey	0.88**	(0.79, 0.97)	0.79***	(0.67, 0.94)	1.09	(0.69, 1.72)
Other registry areas	0.83***	(0.75, 0.92)	0.70***	(0.58, 0.84)	0.50**	(0.29, 0.86)
State buy-in during diagnosis year						
None	Reference		Reference		Reference	
1–6months	0.45***	(0.36, 0.55)	0.37***	(0.25, 0.56)	0.18**	(0.04, 0.70)
7–12months	0.88**	(0.81, 0.96)	0.88	(0.74, 1.03)	0.90	(0.61, 1.35)
Charlson comorbidity index						
0	Reference		Reference		Reference	
1	1.00	(0.94, 1.06)	0.95	(0.85, 1.06)	1.07	(0.82, 1.41)
2+	0.82***	(0.75, 0.89)	0.76***	(0.65, 0.89)	0.62**	(0.40, 0.96)
Poor performance indicators						
No	Reference		Reference		Reference	
Yes	0.78***	(0.71, 0.86)	0.64***	(0.53, 0.78)	0.51**	(0.30, 0.87)
Surgery of PS						
No	Reference		Reference		Reference	
Yes	1.65***	(1.55, 1.76)	2.05***	(1.82, 2.30)	2.13***	(1.58, 2.88)
Surgery of RDS						
No	Reference		Reference		Reference	
Liver metastases surgery	1.17***	(1.10, 1.25)	1.11*	(0.98, 1.26)	0.94	(0.66, 1.35)
Other surgery	1.03	(0.92, 1.14)	0.88	(0.71, 1.09)	1.19	(0.66, 1.35)

SEER, Surveillance, Epidemiology and End Results; PS, primary site; RDS, regional or distal site. Other registry areas: Atlanta, Hawaii, New Mexico, Rural Georgia, San Francisco, San Jose, Utah.

Note: ***, **, and * represented 1%, 5%, and 10% significance levels respectively.

Our analysis also explored factors associated with advancing to second-line treatment and subsequent-line treatment. Surgery of the primary site and being married were associated with receipt of second-line treatment (RR: 2.05, *P* < 0.0001 and RR: 1.46, *P* < 0.0001, respectively). Consistent with prior literature, older individuals and nonwhite individuals were less likely to receive second-line treatment or subsequent-line treatment (Table 2). Compared with individuals living in the Connecticut SEER-region, patients living in other regions (except Los Angeles) were less likely to receive advanced lines of chemotherapy. There was no statistically significant difference between patients residing in the Connecticut and Los Angeles registry areas with regard to the likelihood of receipt of either first-line or second-line treatment. Subsequent-line treatment was only statistically significant for the Kentucky region and for the combined other registry areas. As compared to individuals who did not have state buy-in during their year of diagnosis, those who had less than 12 months of state buy-in were less likely to advance onto second-line treatment or subsequent-line treatment, though this was not statistically significant for those with 7–12 months of state buy-in (1–6 months of buy-in, second-line treatment RR: 0.37, *P* < 0.0001, subsequent-line treatment RR: 0.18, *P*-value: 0.01; 7–12 months of buy-in, second-line treatment RR: 0.88, *P*-value: 0.11, subsequent-line treatment RR: 0.90, *P*-value: 0.62). Statistically significant clinical variables indicative of not receiving second-line treatment or subsequent-line treatment were CCI of two or more and having poor performance status indicators.

## Discussion

For patients with mCC, the standard of care is chemotherapy with improvement in overall survival and quality of life [11, 24–28]. However, treatment guidelines do not differentiate the treatment of younger patients from elderly patients who may be at a greater risk for adverse events. The literature demonstrates that age-related disparities in colon cancer treatment exist. Despite the fact that the median age of colon cancer diagnosis is 71 years, elderly individuals diagnosed with colon cancer are often underrepresented in randomized clinical trials [25, 26]. The only prospective phase III trial (AVEX Trial) conducted in patients diagnosed with mCC whose age was 70 years and greater reported and confirmed the benefit and tolerance of chemotherapy in this group of elderly patients [27].

Our finding of 41% of the examined elderly population receiving therapy is not consistent with practitioners’ impression of receipt of chemotherapy in this population. However, and to our knowledge, this study is the first to explore factors associated with receipt of first-, second-, and subsequent lines of chemotherapy/biologics treatment among elderly patients diagnosed with mCC. Our results suggest that increased age (i.e., greater than 80 years) is the strongest correlate of not advancing through treatment among elderly mCC patients. The literature provides ample evidence that increasing age is associated with decreased organ function, particularly of the liver and kidneys. These two organs are important to drug metabolism and clearance and their decreased function can slow drug metabolism, potentially subjecting the patient to increased drug toxicity [28, 29]. Furthermore, aging is associated with diminished bone marrow reserve, which places elderly patients at an increased risk for chemotherapy-related cytopenias [30]. Many elderly mCC patients may have multiple comorbidities (i.e., cardiovascular disease and hypertension) that compound natural cardiovascular aging. Decreasing cardiovascular health can exacerbate cardiotoxicities associated with some chemotherapy regimen [31]. However, some elderly patients with mCC may continue to treat their multiple comorbidities unaware of the increased risks associated with their chemotherapy [32]. The potential for increased toxicity or concern over the effectiveness of chemotherapy may be a reason why almost 60% of patients in this study do not receive treatment. Potentially, the patient prefers to maintain quality of life as compared to an incrementally smaller quantity of life. It is also expected that some elderly patients did not receive or advance through chemotherapy treatment lines secondary to possible postoperative complications. Many factors (i.e., genetic makeup, age, comorbidities, co-medication, CCI level) can influence how a person's body react to a treatment. For the individuals who received treatment, certain patient-level factors can be ascertained from this dataset but certain factors cannot (i.e., genotype). It is important to note that individuals in this heterogeneous cohort who did not receive treatment could refuse treatment, have contraindications to the recommended treatment, or lacked the skills, support, and services necessary to seek further medical attention; none of which can be assessed by a health care claims dataset but rather by primary data such as patient and provider interviews.

Another important clinical finding was that surgery of the primary site was the only significant characteristic for receiving first-line chemotherapy and advancing to second- and subsequent-line treatments. This may be related to the fact that elderly patients undergoing surgery are likely to have better performance status, younger physiological age with less comorbid conditions, small metastatic tumor burden, resectable metastasis, biologically favorable tumor, and/or better tolerance of chemotherapy.

From a demographic standpoint, marital status was the most significant correlate for receiving first- and second-line chemotherapy. Marital status has been identified in prior studies to be associated with receipt of cancer treatment [33, 34]; however, its role in the models of advancing chemotherapy/biologics has not been investigated. Individuals considered eligible for both Medicare and Medicaid, or having state buy-in during mCC diagnosis year, for less than 6 months were statistically significant less likely to receive treatment. To explain these results, it is possible that individuals who were able to maintain long-term dual eligible status were more successful at accessing care and pursuing treatment than those who were identified with transient, or short-term, dual eligibility. Interestingly, receipt of subsequent lines of treatment was significantly affected by SEER-registry area. For these analyses, we used the Connecticut registry area as the reference group. Patients in most other registry areas were less likely to have advanced chemotherapy treatment as compared to the Connecticut area. Our rationale for this is that the Connecticut is a geographically small, densely populated area with one predominate academic health care system that strongly influences treatment standards for the rest of the area.

This study has several limitations, the primary of which is that it is observational in nature and heavily relies upon an established algorithm to determine line of treatment in the SEER-Medicare population [22]. This algorithm, however, was extensively vetted among clinicians and health services researchers for face validity and internal validity. An additional limitation is that the SEER-Medicare dataset does not include timing of surgical predictors to lines of treatment. Dates of service are available with claims but these are subject to accuracy of when a claim was submitted. Furthermore, this dataset used for these analyses was not constructed to identify time-to-a-specific event as the aim of this study was to identify predictors of chemotherapy treatment. Hence, we were able to identify characteristics associated with receipt of chemotherapy treatment, not to determine the timing around when a specific patient or patient group receives a specific treatment. This study provides a snapshot of the “real world” use of chemotherapy treatment of mCC but it cannot elucidate the role of the physician in the trajectory of the patient's treatment selection of how aggressively treatment is pursued. Using our published algorithm, we were able to examine lines of treatment and did not have a surrogate indicator that could capture the role of the provider in treatment. To ascertain why a provider and a patient make observed treatment decisions reflected in administrative claims, a researcher must discuss decision with the patient and the provider. Very little information exists to guide patients and practitioners about the factors that influence treatment or the additional therapy used to treat diagnosed mCC.

Although commonly grouped together, this study did not include patients diagnosed with rectal cancer (RC). This was done intentionally because of the differences between colon cancer and RC. This study examined the treatment of mCC which is treated with surgery and a combination of chemotherapy and biologics. RC is treated similarly but radiation treatment is also a large cornerstone for its treatment, unlike colon cancer. Additionally, monoclonal antibodies are not approved to treat RC. Among the therapies approved for the use of treatment of colon cancer capecitabine, oxaliplatin, and leucovorin are not approved for the treatment of RC, although the combination 5-FU/leucovorin is approved for initial treatment of advanced colorectal cancer [35]. Another characteristic that makes this study unique to the mCC population is that some surgical information and geographic registry site were included. Typically, observational studies do not contain clinical, demographic, and administrative claims information, and as a result of the combination of this information in the SEER-Medicare dataset, we were able to obtain more granularity of characteristics that correlate with receipt of treatment for elderly mCC patients.

## Conclusion

This study demonstrated that a large portion of elderly mCC patients do not receive treatment. Among those who receive treatment, the strongest correlates of advancing through treatment lines are having surgery of the primary site and being married. Physicians and clinical decision makers should focus on individualizing the treatment of older patients based on their ability to tolerate surgery and chemotherapy, age, performance status, and comorbid conditions while also considering the goals of therapy. Additionally, resources should be available to individuals with short-term state buy-in to allow them to access care.

## References

[1] Van Cutsem E, Tejpar S, Vanbeckevoort D, Peeters M, Humblet Y, Gelderblom H (2012). Intrapatient cetuximab dose escalation in metastatic colorectal cancer according to the grade of early skin reactions: the randomized EVEREST study. J. Clin. Oncol.

[2] Douillard JY, Siena S, Cassidy J, Tabernero J, Burkes R, Barugel M (2010). Randomized, phase III trial of panitumumab with infusional fluorouracil, leucovorin, and oxaliplatin (FOLFOX4) versus FOLFOX4 alone as first-line treatment in patients with previously untreated metastatic colorectal cancer: the PRIME study. J. Clin. Oncol.

[3] Peeters M, Price TJ, Cervantes A, Sobrero AF, Ducreux M, Hotko Y (2010). Randomized phase III study of panitumumab with fluorouracil, leucovorin, and irinotecan (FOLFIRI) compared with FOLFIRI alone as second-line treatment in patients with metastatic colorectal cancer. J. Clin. Oncol.

[4] Cassidy J, Clarke S, Díaz-Rubio E, Scheithauer W, Figer A, Wong R (2008). Randomized phase III study of capecitabine plus oxaliplatin compared with fluorouracil/folinic acid plus oxaliplatin as first-line therapy for metastatic colorectal cancer. J. Clin. Oncol.

[5] Saltz LB, Clarke S, Díaz-Rubio E, Scheithauer W, Figer A, Wong R (2008). Bevacizumab in combination with oxaliplatin-based chemotherapy as first-line therapy in metastatic colorectal cancer: a randomized phase III study. J. Clin. Oncol.

[6] Falcone A, Ricci S, Brunetti I, Pfanner E, Allegrini G, Barbara C (2007). Phase III trial of infusional fluorouracil, leucovorin, oxaliplatin, and irinotecan (FOLFOXIRI) compared with infusional fluorouracil, leucovorin, and irinotecan (FOLFIRI) as first-line treatment for metastatic colorectal cancer: the Gruppo Oncologico Nord Ovest. J. Clin. Oncol.

[7] Fuchs CS, Marshall J, Mitchell E, Wierzbicki R, Ganju V, Jeffery M (2007). Randomized, controlled trial of irinotecan plus infusional, bolus, or oral fluoropyrimidines in first-line treatment of metastatic colorectal cancer: results from the BICC-C Study. J. Clin. Oncol.

[8] Van Cutsem E, Peeters M, Siena S, Humblet Y, Hendlisz A, Neyns B (2007). Open-label phase III trial of panitumumab plus best supportive care compared with best supportive care alone in patients with chemotherapy-refractory metastatic colorectal cancer. J. Clin. Oncol.

[9] Fuchs C, Moore M, Harker G, Villa L, Rinaldi D, Hecht JR (2003). Phase III comparison of two irinotecan dosing regimens in second-line therapy of metastatic colorectal cancer. J. Clin. Oncol.

[10] Jäger E, Heike M, Bernhard H, Klein O, Bernhard G, Lautz D (1996). Weekly high-dose leucovorin versus low-dose leucovorin combined with fluorouracil in advanced colorectal cancer: results of a randomized multicenter trial. Study Group for Palliative Treatment of Metastatic Colorectal Cancer Study Protocol 1. J. Clin. Oncol.

[11] Mullins CD, Hsiao F, Onukwugha E, Pandya N, Hanna N (2012). Comparative and cost-effectiveness of oxaliplatin-based or irinotecan-based regimens compared with 5-fluorouracil/leucovorin alone among US elderly stage IV colon cancer patients. Cancer.

[12] National Comprehensive Cancer Network (2013). http://www.nccn.org/professionals/physician_gls/pdf/colon.pdf.

[13] Boland G, Chang G, Haynes A, Chiang YJ, Chagpar R, Xing Y (2013). Association between adherence to National Comprehensive Cancer Network treatment guidelines and improved survival in patients with colon cancer. Cancer.

[14] Chagpar R, Xing Y, Chiang YJ, Feig BW, Chang GJ, You YN (2012). Adherence to stage-specific treatment guidelines for patients with colon cancer. J. Clin. Oncol.

[15] Hung A, Mullins CD (2013). Relative effectiveness and safety of chemotherapy in elderly and nonelderly patients with stage III colon cancer: a systematic review. Oncologist.

[16] McCleary N, Odejide O, Szymonifka J, Ryan D, Hezel A, Meyerhardt J (2013). Safety and effectiveness of oxaliplatin-based chemotherapy regimens in adults 75 years and older with colorectal cancer. Clin. Colorectal Cancer.

[17] Sundararajan V, Hershman D, Grann VR, Jacobson JS, Neugut AI (2002). Variations in the use of chemotherapy for elderly patients with advanced ovarian cancer: a population-based study. J. Clin. Oncol.

[18] Kahn KL, Adams JL, Weeks JC, Chrischilles EA, Schrag D, Ayanian JZ (2010). Adjuvant chemotherapy use and adverse events among older patients with stage III colon cancer. JAMA.

[19] Jessup JM, Stewart A, Greene FL, Minsky BD (2005). Adjuvant chemotherapy for stage III colon cancer: implications of race/ethnicity, age, and differentiation. JAMA.

[20] Potosky AL, Harlan LC, Kaplan RS, Johnson KA, Lynch CF (2002). Age, sex, and racial differences in the use of standard adjuvant therapy for colorectal cancer. J. Clin. Oncol.

[21] National Cancer Institute (2012). http://seer.cancer.gov/about/overview.html.

[22] Bikov K, Mullins CD, Seal B, Onukwugha E, Hanna N (2013). Algorithm for identifying chemotherapy/biological regimens for metastatic colon cancer in SEER-Medicare. Med. Care.

[23] Zou G (2004). A modified poisson regression approach to prospective studies with binary data. Am. J. Epidemiol.

[24] Douillard JY, Cunningham D, Roth AD, Navarro M, James RD, Karasek P (2000). Irinotecan combined with fluorouracil compared with fluorouracil alone as first-line treatment for metastatic colorectal cancer: a multicentre randomised trial. Lancet.

[25] American Cancer Society (2011). Global cancer fact & figures.

[26] American Cancer Society (2011). http://www.cancer.org/acs/groups/content/@epidemiologysurveilance/documents/document/acspc-028323.pdf.

[27] Cunningham D, Lang I, Lorusso V, Ocvirk J, Shin D, Jonker DJ (2012). Bevacizumab (bev) in combination with capecitabine (cape) for the first-line treatment of elderly patients with metastatic colorectal cancer (mCRC): results of a randomized international phase III trial (AVEX). J. Clin. Oncol.

[28] Sawhney R, Sehl M, Naeim A (2005). Physiologic aspects of aging: impact on cancer management and decision making, part I. Cancer J.

[29] Sehl M, Sawhney R, Naeim A (2005). Physiologic aspects of aging: impact on cancer management and decision making, part II. Cancer J.

[30] Foran JM, Shammo JM (2012). Clinical presentation, diagnosis, and prognosis of myelodysplastic syndromes. Am. J. Med.

[31] Scappaticci FA, Skillings JR, Holden SN, Gerber HP, Miller K, Kabbinavar F (2007). Arterial thromboembolic events in patients with metastatic carcinoma treated with chemotherapy and bevacizumab. J. Natl. Cancer Inst.

[32] Cashman J, Wright J, Ring A (2010). The treatment of co-morbidities in older patients with metastatic cancer. Support. Care Cancer.

[33] Wang L, Wilson SE, Stewart DB, Hollenbeak CS (2011). Marital status and colon cancer outcomes in US Surveillance, Epidemiology and End Results registries: does marriage affect cancer survival by gender and stage?. Cancer Epidemiol.

[34] Kutner JS, Vu KO, Prindiville SA, Byers TE (2000). Patient age and cancer treatment decisions. Patient and physician views. Cancer Pract.

[35] National Cancer Institute (2013). http://www.cancer.gov/cancertopics/druginfo/fda-oxaliplatin.

